# Optimized allotopic expression of mitochondrial ND6 transgene restored complex I and apoptosis deficiencies caused by LHON-linked ND6 14484T > C mutation

**DOI:** 10.1186/s12929-023-00951-1

**Published:** 2023-08-03

**Authors:** Jing Wang, Yanchun Ji, Cheng Ai, Jia-Rong Chen, Dingyi Gan, Juanjuan Zhang, Jun Q. Mo, Min-Xin Guan

**Affiliations:** 1 Center for Mitochondrial Biomedicine, The Fourth Affiliated Hospital, Zhejiang University School of Medicine, Hangzhou, Zhejiang China; 2 Division of Medical Genetics and Genomics, The Children’s Hospital, Zhejiang University School of Medicine and National Clinical Research Center for Child Health, Hangzhou, Zhejiang China; 3 Institute of Genetics, Zhejiang University School of Medicine, 866 Yuhangtang Road, Hangzhou, Zhejiang China; 4 School of Optometry and Ophthalmology and Eye Hospital, Wenzhou Medical University, Wenzhou, Zhejiang China; 5 Department of Pathology, Rady Children’s Hospital, University of California at San Diego School of Medicine, San Diego, California USA; 6 Zhejiang Provincial Key Laboratory of Genetic and Developmental Disorders, Hangzhou, Zhejiang China; 7 Key Lab of Reproductive Genetics, Ministry of Education of PRC, Zhejiang University, Hangzhou, Zhejiang China

**Keywords:** Leber’s hereditary optic neuropathy, Mitochondrial DNA mutation, Complex I, Allotopic expression, Apoptosis, Mitophagy

## Abstract

**Background:**

Leber’s hereditary optic neuropathy (LHON) is a maternally inherited eye disease due to mutations in mitochondrial DNA. However, there is no effective treatment for this disease. LHON-linked ND6 14484T > C (p.M64V) mutation caused complex I deficiency, diminished ATP production, increased production of reactive oxygen species (ROS), elevated apoptosis, and impaired mitophagy. Here, we investigated if the allotopic expression of human mitochondrial ND6 transgene corrected the mitochondrial dysfunctions due to LHON-associated m.14484T > C mutation.

**Methods:**

Nucleus-versions of ND6 was generated by changing 6 non-universal codons with universal codons and added to mitochondrial targeting sequence of COX8. Stable transfectants were generated by transferring human ND6 cDNA expressed in a pCDH-puro vector into mutant cybrids carrying the m.14484T > C mutation and control cybrids. The effect of allotopic expression of ND6 on oxidative phosphorylation (OXPHOS) was evaluated using Blue Native gel electrophoresis and extracellular flux analyzer. Assessment of ROS production in cell lines was performed by flow cytometry with MitoSOX Red reagent. Analyses for apoptosis and mitophagy were undertaken via flow cytometry, TUNEL and immunofluorescence assays.

**Results:**

The transfer of human ND6 into the cybrids carrying the m.14484T > C mutation raised the levels of ND6, ND1 and ND4L but did not change the levels of other mitochondrial proteins. The overexpression of ND6 led to 20~23% increases in the assembly and activity of complex I, and ~ 53% and ~ 33% increases in the levels of mitochondrial ATP and ΔΨm in the mutant cybrids bearing m.14484T > C mutation. Furthermore, mutant cybrids with overexpression of ND6 exhibited marked reductions in the levels of mitochondrial ROS. Strikingly, ND6 overexpression markedly inhibited the apoptosis process and restored impaired mitophagy in the cells carrying m.14484T > C mutation. However, overexpression of ND6 did not affect the ND6 level and mitochondrial functions in the wild-type cybrids, indicating that this ND6 level appeared to be the maximum threshold level to maintain the normal cell function.

**Conclusion:**

We demonstrated that allotopic expression of nucleus-versions of ND6 restored complex I, apoptosis and mitophagy deficiencies caused by the m.14484T > C mutation. The restoration of m.14484T > C mutation-induced mitochondrial dysfunctions by overexpression of ND6 is a step toward therapeutic interventions for LHON and mitochondrial diseases.

## Introduction

Leber’s hereditary optic neuropathy (LHON) is the most common maternally inherited eye disease that presents with the loss of central vision in young adults, due to the degeneration of retinal ganglion cells and their axons [1–6]. The majority of LHON cases globally results from one of three mitochondrial DNA (mtDNA) mutations (ND1 3460G > A, ND4 11778G > A, and ND6 14484T > C), which affects the essential subunits of complex I (NADH: ubiquinone oxidoreductase) [7–13]. These mtDNA mutations resulted in the complex I deficiency, diminished ATP synthesis and an increasing generation of reactive oxygen species (ROS) [14–20]. Of these, the m.14484T > C mutation changed a highly conserved methionine at position 64 with valine (p.M64V) in ND6, thereby perturbing the structure and function of complex I [20]. In fact, the M64 forms a nonpolar interaction Y59 in the ND6, Y59 in the ND6 interacts with E34 of ND4L, and L60 of ND6 interacts with the Y114 of ND1 [20, 21]. Therefore, the m.14484T > C (p.M64V) mutation caused the reductions in the levels of ND6, ND1 and ND4L [20]. Furthermore, mutant cell lines bearing the m.14484T > C mutation conferred decreased activity of complex I, respiratory deficiency, diminished mitochondrial ATP production, reduced membrane potential, and increased production of ROS in the mutant cybrids [20]. The m.14484T > C mutation-induced alterations promoted apoptosis and impaired PINK1/Parkin-dependent mitophagy [20]. Subsequently, the energy failure and increasing oxidative stress may lead to the degeneration of retinal ganglion cells including defects in neuronal differentiation, morphology and electrophysiological properties [5, 6, 22]. However, the pathogenic mechanism underlying LHON-associated mtDNA mutations is still not well understood and some technical challenges such as transferring exogenous genes into mitochondrial genomes and lacking the precise nucleotide editing by CRISPR-based genome editing technology hampered the development of effective treatment for this disease [23–26].

Allotopic expression of mitochondrial genes that deliberated functional relocation of mitochondrial genes into the nucleus, followed by import of the gene-encoded polypeptide from the cytoplasm into the mitochondria is a promising therapeutic approach to treat the LHON [25–30]. In particular, the sequences of ND1 or ND4 genes were recoded into the universal code (U-code) to permit the correct translation of their mRNA in the cytoplasm and ultimately delivered in functional proteins to mitochondria to rescue the mitochondrial deficiency due to m.11778G > A or m.3460G > A mutation [30–33]. Therefore, optimized allotopic expression of mitochondrial ND6 transgene may restore the complex I and apoptosis deficiencies due to LHON-linked m.14484T > C mutation. In this investigation, we modified the mtDNA-encoded ND6 gene by altering the mtDNA code to the U-code for the correct synthesis of ND6 polypeptide in cytosol, and added a mitochondrial targeting sequence (MTS) with first 25 amino acids of COX8 for delivery of nucleus-version of ND6 into mitochondria [34]. We then constructed stable transfectants by transferring modified ND6 cDNA into mutant cybrids carrying the m.14484T > C mutation and control cybrids lacking the mutation [20]. The stable transfectants were than analyzed for the levels of mitochondrial proteins and complex I activity, oxygen consumption rate (OCR), the levels of cellular and mitochondrial ATP as well as mitochondrial membrane potential. These transfectants were further evaluated for the effects of ND6 transgene on the production of ROS, apoptosis process and mitophagy.

## Materials and methods

### Plasmid construction

**Fig. 1:**
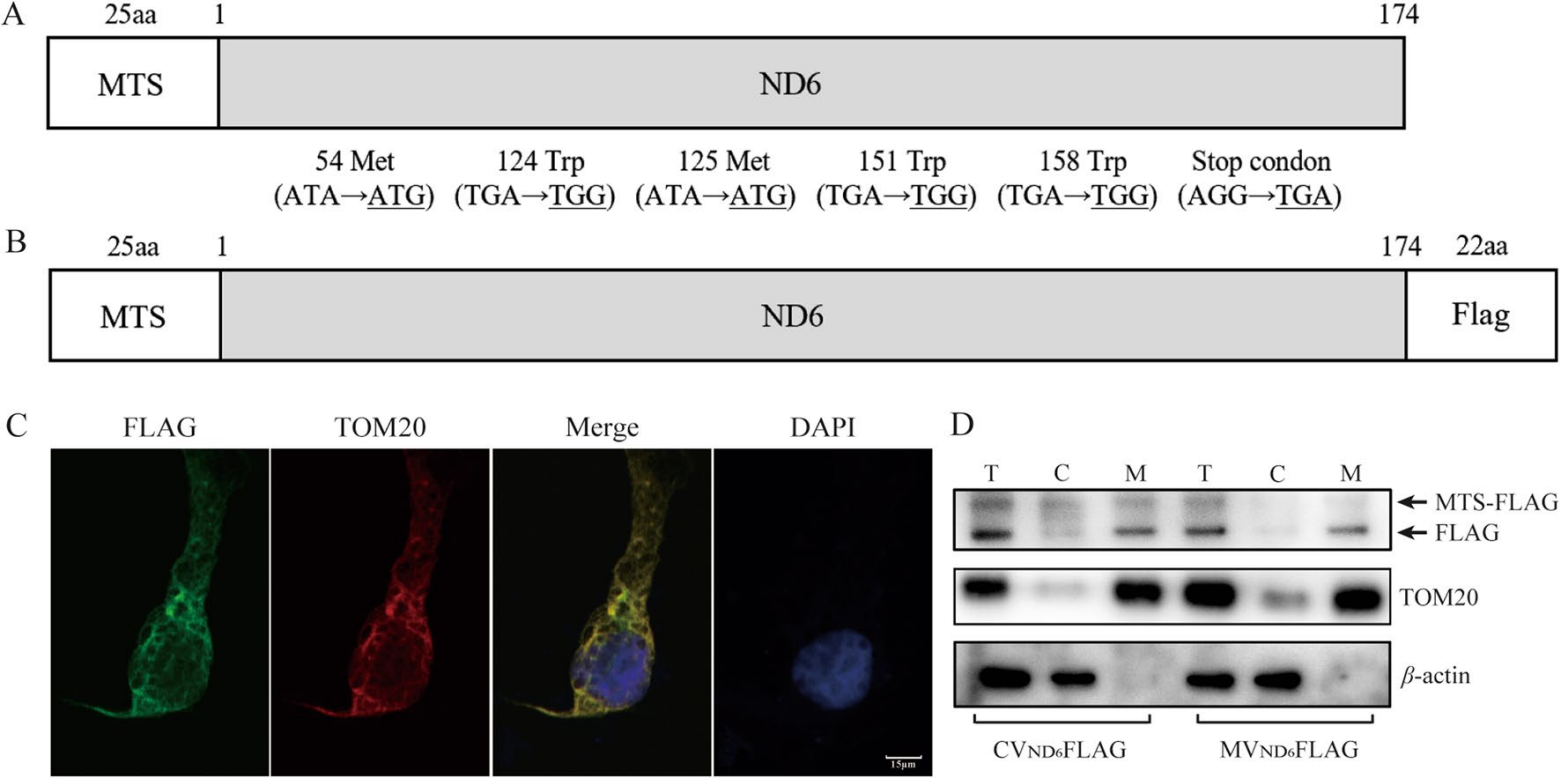
Subcellular location of modified human ND6. **A** Scheme for the structure of nucleus-versions of human ND6. Six codons in mtDNA encoding ND6 were modified as universal codons. MTS of COX8 with 25  amino acids was added to initiating codon of ND6. **B** A carboxy terminus FLAG-tagged nucleus-versions of human ND6. **C** Subcellular localization of nucleus-versions human ND6 by immunofluorescence in control cybrids. FLAG-tagged ND6 (shown in green), TOM20 (nucleus-encoding mitochondrial membrane) (shown in red). Scale bars, 15 μm. **D** Subcellular localization of WT and MT nucleus-versions human ND6 by Western blotting with anti-FLAG, TOM20 and β-actin (cytosol). T, total cell lysate; C, cytosol; M, mitochondria

DNA fragments spanning human mitochondrial ND6 open reading frame (ORF) with the wild type (WT) and mutant (MT) versions were synthesized from Tsingke Biotechnology Corp (Beijing, China). ND6 ORFs of 522 nucleotides (174 amino acids) were recoded for the 6 non-universal codons and optimized as universal codons to achieve a high-level expression in human cells [29] (Fig. 1A). COX8 MTS bearing 75 nucleotides coding for 25 amino acids was appended to the ND6 sequence in frame with the ND6 AUG codon and result constructs named as ND6-COX8 contained the *EcoR*I and *Not*I digestion sites that allowed the insertion of the pCDH-puro vector (Addgene, Watertown, MA) [32–35]. Furthermore, the constructs named ND6-COX8-FLAG were produced by the synthesis of above recoded ND6 proteins with a Flag epitope appended to the C-terminal region. These constructs were confirmed by Sanger sequence analysis for accuracy of the COX8-ND6 and COX8-ND6Flag sequences (Tsingke Biotechnology Ltd.).

### Construction of stable transfectants

Mutant cybrids (M) carrying the m.14484T > C mutation, and control cybrids (C) belonging to the same mtDNA haplogroup D4 but lacking the mutation were grown in DMEM (containing 4.5 mg of glucose and 0.11 mg of pyruvate per ml), supplemented with 5% fetal bovine serum (FBS) [20]. The resultant constructs or vector only were transfected into M and C cell lines using the jetPRIMETM transfection reagent (Polyplus Transfection, Graffenstaden, France) according to the manufacturer’s protocol. The stable transfectants were isolated by culturing cells in DMEM supplemented with 1 μg/ml puromycin and 10% FBS for 2 weeks. The resultant clones were examined for the expression of ND6 by immunofluorescence analysis and Western blot analysis [36]. However, these stable transfectants after growing for another 4 weeks tended to substantially increase the cell death.

### Subcellular localization of human ND6

The COX8-ND6-Flag plasmids were transfected into control cybrid cells using the jetPRIMETM transfection reagent (Polyplus Transfection) according to the manufacturer’s protocol. Immunofluorescence assays were performed as detailed elsewhere [36]. The over-expression of ND6-FLAG fusion protein in the mutant and control cells was further verified by Western blot analysis using total lysate and mitochondrial and cytosol fractions with anti-FLAG, TOM20 and β-actin, respectively [37].

### Western blot analysis

Western blot analysis was undertaken as detailed elsewhere [38]. Twenty micrograms of total cellular proteins were electrophoresed through 10% bis–Tris SDS–polyacrylamide gels, transferred to a polyvinyl difluoride (PVDF) membrane (Thermo Fisher Scientific, Shanghai, China) and then processed for immunoblotting. The first antibodies used for this investigation were from Proteintech [*β*-actin (66009-1-Ig), ND1 (19703-1-AP), ND5 (55410-1-AP), NDUFS1 (12444-1-AP), NUDFB8 (14794-1-AP), SDHB (10620-1-AP), COX5A (11448-1-AP), BAX (60267-1-Ig), Caspase 3 (19677-1-AP), and Caspase 9 (10380-1-AP)], ABclonal Technology [ND6 (A17991), NDUFA10 (A10123), NDUFS5 (A1265), NDUFC2 (A15073), NDUFA11 (A16239), NDUFS2 (A12858), Cytochrome *C* (A4912), SOD1 (A12537), SOD2 (A19576), Catalase (A11220), TOM20(A19403), FLAG (AE005), LC3B (A7198), PINK1 (A7131), Parkin (A0968), and P62 (A11250)], Abcam Biotechnology [ND3 (ab192306)], Abcepta [ND4L (AP17147b)], and Cell Signaling Technology [Bcl-xL (2762S) and Caspase 7 (12827)], respectively. The secondary antibodies used for these assays were from Beyotime Biotechnology (peroxidase AffiniPure goat anti-rabbit IgG [A0208] and goat anti-mouse IgG [A0216]), respectively. The protein signals were detected using the ECL system (MilliporeSigma, Burlington, MA). Quantification of density in each band was performed as detailed previously [38].

### Blue native (BN) gel electrophoresis and in-gel activity assays

Mitochondria were isolated from various cell lines by following the protocol, as described elsewhere [39]. Blue native gel electrophoresis and in-gel activity assays were performed by using mitochondrial proteins isolated from cell lines, as detailed elsewhere [40, 41].

### Measurements of oxygen consumption

The oxygen consumption rate (OCR) in various cell lines were measured with a Seahorse Bioscience XF-96 extracellular flux analyzer (Seahorse Bioscience), as detailed previously [42]. Cells from each cell line were seeded at a density of 2 × 10^4^ cells per well on Seahorse XF96 polystyrene tissue culture plates (Seahorse Bioscience, North Billerica, Massachusetts). Inhibitors for various OXPHOS complexes were used at the following concentrations: 1 μM of oligomtcin (to inhibit the ATP synthase), 0.5  μM of carbonyl cyanide p-trifluoromethoxyphenylhydrazone (FCCP) (to uncouple the mitochondrial inner membrane and allow for maximum electron flux through the ETC), 1 μM of rotenone (to inhibit complex I), and 1 μM of antimycin A (to inhibit complex III), respectively.

### ATP measurements

The cellular and mitochondrial ATP levels were analyzed by a Cell Titer-Glo luminescent cell viability assay kit (Promega) according to the modified procedures of the manufacturer, as described previously [38].

### Assessment of mitochondrial membrane potential

Mitochondrial membrane potential was measured with a JC-10 Mitochondrial Membrane Potential Assay Kit (ab112133, Abcam, Cambridge, United Kingdom), according to the manufacturer’s general recommendations, with some modifications as detailed elsewhere [38, 43].

### ROS measurements

The levels of mitochondrial ROS generation were examined with MitoSOX assay, as detailed previously [36, 44].

### Annexin V/PI apoptosis assay by flow cytometry

For discrimination of apoptotic and non-apoptotic cells by Annexin V/PI staining, cells were harvested and stained with Annexin V and 1 μL of propidium iodide (PI) (V13242, Thermo Fisher Scientific, Waltham, MA) according to the manufacturer’s instruction, as described elsewhere [17, 45]. Each sample was detected by NovoCyte (Agilent Technologies, San Diego, California) and analyzed using NovoExpress software.

### Tunnel assay

The TUNEL assay was carried out using the One Step TUNEL Apoptosis Assay Kit (C1086, Beyotime, Shanghai, China) according to the manufacturer’s protocol with some modifications, as detailed elsewhere [45].

### Immunofluorescence analysis

Immunofluorescence experiments were performed as described previously [45, 46]. Cells were cultured on cover glass slips (Thermo Fisher), fixed in 4% formaldehyde for 15 min, permeabilized with 0.2% Triton X-100, blocked with 5% FBS for 1 h, and immunostained with Parkin or LAMP1antibody overnight at 4 °C. The cells were then incubated with Alex Fluor 488 goat anti-mouse IgG (H + L) (Thermo Fisher), stained with MitoTracker Red (Thermo Fisher) for 20 min and with 4', 6-diamidino-2-phenylindole (DAPI) (Thermo Fisher) for 15 min, and mounted with Fluoromount (Sigma-Aldrich, St. Louis, MO). Cells were examined using a confocal fluorescence microscope (Olympus Fluoview FV3000, Japan) with three lasers (Ex/Em = 550/570 nm, 492/520 nm and 358/461 nm).

### Statistical analysis

All statistical analyses were performed using the unpaired, two-tailed Student’s *t-*test contained in the GraphPad Prism 8 program (GraphPad Software) and Microsoft Excel (Version 2019). In all graphs, error bars displayed on graphs represent means ± standard error of the mean (SEM) of at least three independent experiments. Values of *P < 0.05, **P < 0.01 and ***P < 0.001 were considered to be statistically significant.

## Results

### Construction of stable transfectants expressing the human ND6 transgene

Human ND6 ORF of 522 nucleotides (174 amino acids) was synthesized by replacement of 6 non-universal codons with universal codons for the optimized allotopic expression of ND6 gene (Fig. 1A). Seventy-five nucleotides for the 25 amino acids of COX8 MTS was then appended to the ND6 sequence in frame with ND6 AUG condon (Fig. 1A). Human ND6 cDNA expressed in a pCDH-puro vector or vector only was transfected into the mutant cybrids (M) carrying the m.14484T > C mutation and control cybrids (C) lacking this mutation [20]. Four stable transfectants each [CV0 (vector only in control cybrids), CVND6 (exogenous ND6 in control cybrids), MV0 (vector only in mutant cybrids), and MVND6 (exogenous ND6 in mutant cybrids)] were obtained by culturing cells in DMEM supplemented with 1 μg/ml puromycin and 10% FBS for 2 weeks. However, the stable transfectants grow in the medium with purmycin.

To examine if nucleus-versions of ND6 entered mitochondrion, a FLAG-tagged version of MTS-ND6 was transiently expressed in the control cell line (C) and the mutant cybrid cell line M (Fig. 1B). As shown in Fig. 1C, a carboxy terminus FLAG-tagged MTS-ND6 displayed overlap with the mitochondrial protein TOM20. The subcellular location of nucleus-versions of ND6 was further examined by Western blotting in total lysate and mitochondrial fraction and cytosol with anti-FLAG, TOM20 and β-actin, respectively. As shown in Fig. 1D, cellular fraction experiments of cells using anti-FLAG revealed the two bands corresponding to the ND6 precursor and mature form in lysate, only one band corresponding to mature ND6 form in mitochondrial fraction but no detectable band in cytosol fraction. These results demonstrated that the nucleus-versions of ND6 localized to mitochondria.

### Elevated levels of ND6, ND4L and ND1

**Fig. 2:**
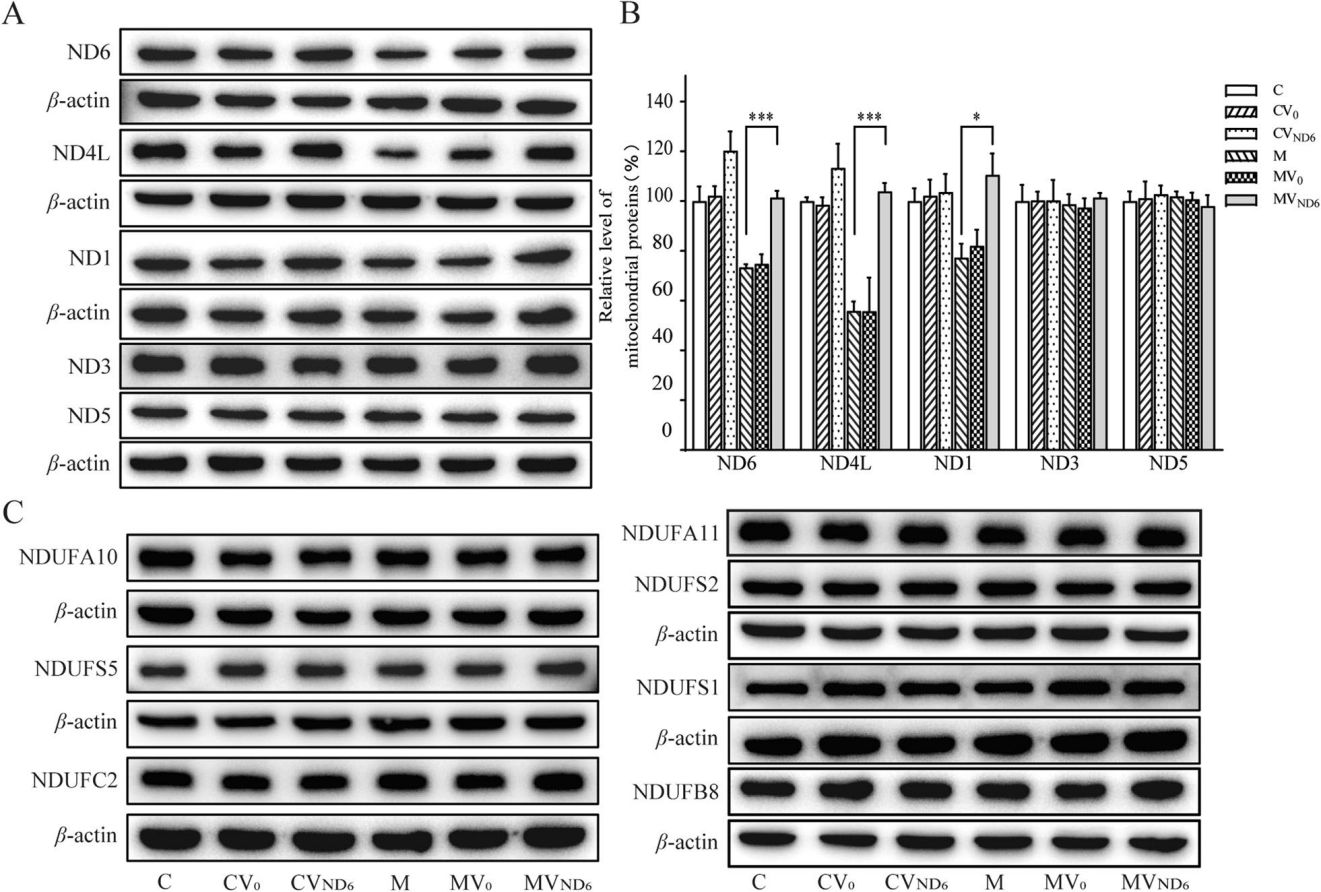
Analysis of complex I subunits encoded by mitochondrial and nuclear genes. **A** Western blot analysis of mtDNA encoding proteins. Twenty μg of total cellular proteins from various cell lines were electrophoresed through a denaturing polyacrylamide gel, electroblotted, and hybridized with ND6, ND4L, ND1, ND3 and ND5 antibodies, with *β*-actin as a loading control. **B** Quantification of ND6, ND4L, ND1, ND3 and ND5 in C, CV0, CVND6, M, MV0, and MVND6 cell lines. The calculations were based on three independent determinations in each cell line. The error bars indicate standard error of the mean (SEM). *P* indicates significance based on Student’s *t*-test of the differences between M and MVND6 cell lines. **C** Western blot analysis of nucleus-encoding complex I subunits. Twenty μg of total cellular proteins from various cell lines were electrophoresed through a denaturing polyacrylamide gel, electroblotted, and hybridized with NDUFA10, NDUFS5, NDUFC2, NDUFA11, NDUFS2, NDUFS1 and NDUFB8 antibodies, with *β*-actin as a loading control

Our previous study showed that mutant cybrids bearing the m.14484T > C mutation exhibited the decreases in the levels of ND6, ND4L and ND1 [20]. We examined the levels of these mitochondrial proteins among various cell lines using Western blot analysis. As shown in Fig. 2A and B, the levels of ND6 in cell lines CV0, CVND6, M, MV0, and MVND6 were 102%, 120%, 73%, 75% and 101% relative to the average values of the parental control cell lines C, respectively. Furthermore, the average levels of ND4L in cell lines CV0, CVND6, M, MV0, and MVND6 were 99%, 113%, 56%, 56%, and 104% relative to the average values of the control cell line C, respectively, while the average levels of ND1 in cell lines CV0, CVND6, M, MV0, and MVND6 were 102%, 104%, 77%, 82%, and 111% relative to the average values of the control cell line C, respectively. However, the levels of ND3 and ND5 in the transfectants were comparable with those in the parental control cell line C.

The ND6 interacts with nucleus-encoding NDUFC2, NDUFS5, NDUFA10 and NDUFA11 of complex I subunits [20, 21]. We measured the levels of NDUFA10, NDUFS5, NDUFC2, NDUFA11, NDUFS2, NDUFS1 and NDUFB8 in these six cell lines using Western blot analysis. As shown in Fig. 2C, there were no significant difference in the levels of these subunits among these six cell lines.

### Raising the activity and assembly of complex I

**Fig. 3:**
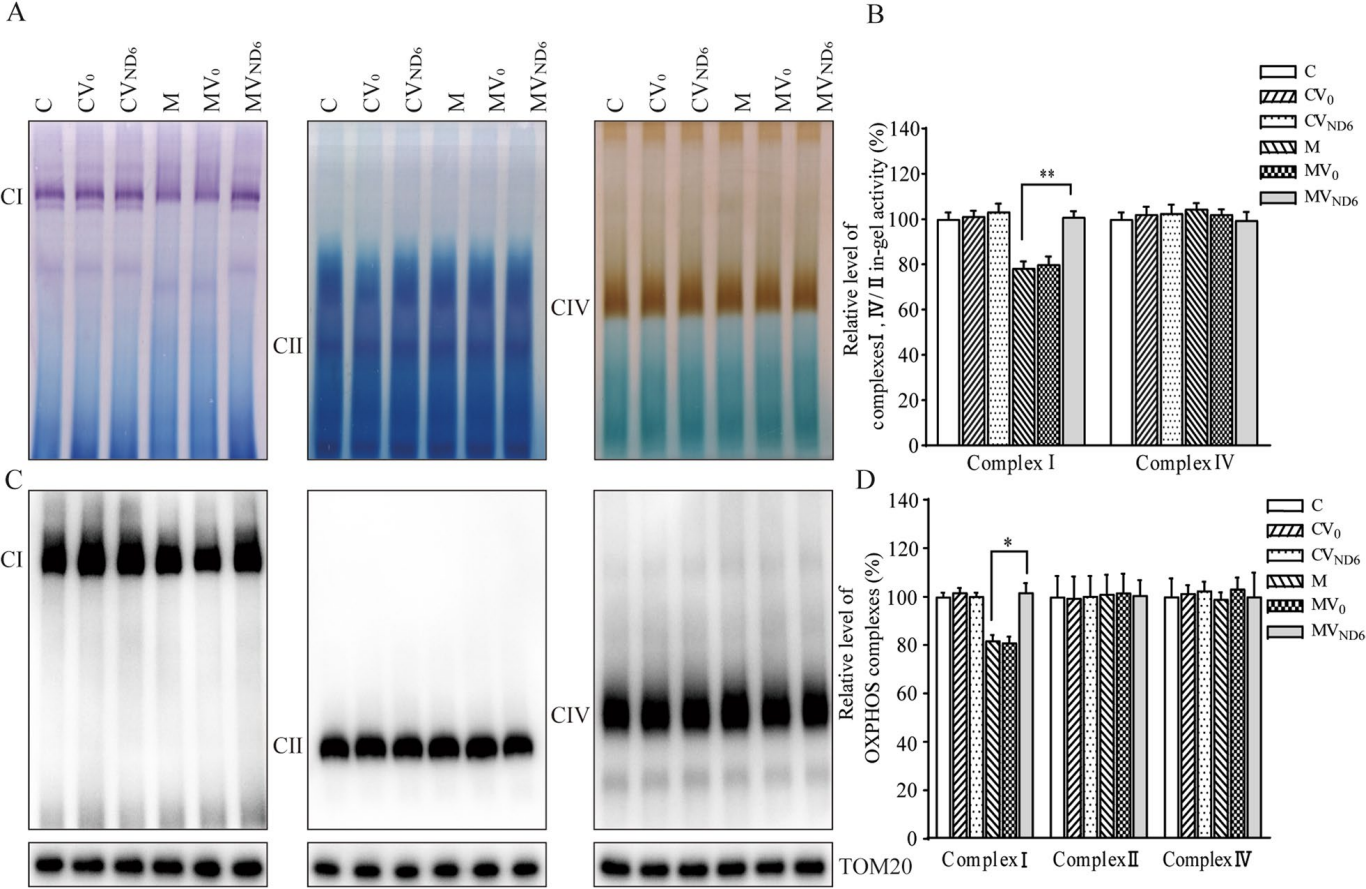
Analysis of OXPHOS complexes. **A** In-gel activity of complexes I, II and IV. The activities of OXPHOS complexes from various cell lines after BN-PAGE were measured in the presence of specific substrates [NADH and NTB for complex I, sodium succinate, phenazine methosulfate, and NTB for complex II, DAB and cytochrome c for complex IV]. **B** Quantification of in-gel activities of complexes I and IV. The calculations were based on three independent determinations in each cell line. **C** The levels of complexes I, II and IV by BN-PAGE. Twenty micrograms of mitochondrial proteins from various cell lines were electrophoresed through a BN- gel, electroblotted and hybridized with antibodies specific for subunits of complexes I, II and IV complexes (NDUFS2 antibody for complex I, SDHB antibody for complex II, and COX5A antibody for complex IV), and with TOM20 as a loading control. **D** Quantification of levels of complexes I, II, and IV. The calculations were based on three independent determinations in each cell line. Graph details and symbols are explained in the legend to Fig. 2

To determine whether the overexpression of ND6 improved the m.14484T > C mutation-induced complex I deficiencies, we assessed if the potential consequence of overexpression of ND6 affected the stability and activity of complex I using the in-gel activity assay. Mitochondrial membrane proteins isolated from the six cell lines were separated by BN-PAGE and stained with specific substrates of complexes I, II, and IV [40, 41]. As shown in Fig. 3A and B, the in-gel activities of complex I in the cell lines CV0, CVND6, M, MV0, and MVND6 were 101%, 103%, 78%, 80% and 101%, relative to the average values of parental control cell line. In contrast, the average in-gel activities of complex IV in these six cell lines were comparable with the control cell line.

Furthermore, we measured the activities of respiratory complexes assembly using BN-PAGE/immunoblot analysis. The mitochondrial membrane proteins isolated from the six cell lines were separated by BN-PAGE, electroblotting, and hybridizing with NDUFS2 (subunit of complex I), SDHB (subunit of complex II), COX5A (subunit of complex IV) and TOM20 as a loading control [41]. As shown in Fig. 3C and D, the levels of complex I in the cell lines CV0, CVND6, M, MV0, and MVND6 were 102%, 100%, 82%, 81% and 102% relative to the average values in the control cell line C. By contrast, the levels of complexes II and IV in other cell lines were comparable to the control cell line C.

**Fig. 4:**
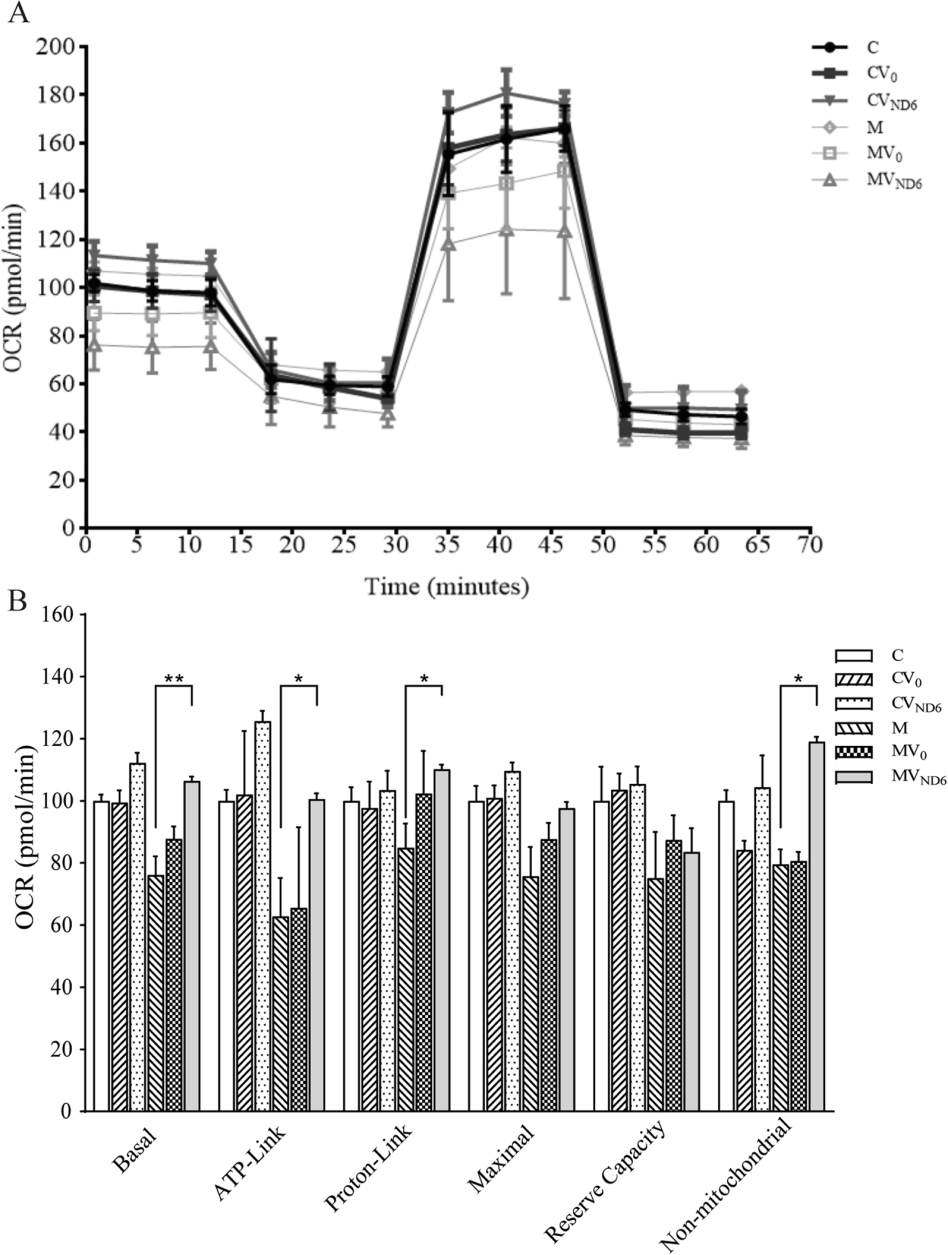
Respiration assays. **A** An analysis of O_2_ consumption in the various cell lines using different inhibitors. The OCRs were first measured on 2 × 10^4^ cells of each cell line under basal conditions and then sequentially added to oligomycin (1.0 µM), FCCP (0.5 µM), rotenone (1.0 µM), and antimycin A (1.0 µM) at the indicated times to determine the different parameters of mitochondrial functions. **B** Graphs show the basal OCR, ATP-linked OCR, proton leak OCR, maximal OCR, reserve capacity, and non-mitochondrial OCR among six cell lines. The non-mitochondrial OCR was determined as the OCR after rotenone/antimycin A treatment. The basal OCR was determined as the OCR before oligomycin minus the OCR after rotenone/antimycin A treatment. The ATP-linked OCR was determined as the OCR before oligomycin minus the OCR after oligomycin. The proton leak OCR was determined as the basal OCR minus the ATP-linked OCR. The maximal OCR was determined as the OCR after FCCP minus the non-mitochondrial OCR. Reserve capacity was defined as the difference between the maximal OCR after FCCP minus the basal OCR. The average values of three independent experiments for each cell line are shown. Graph details and symbols are explained in the legend to Fig. 2

We further measured the OCR of various cell lines via extracellular flux analyzer [42]. As shown in Fig. 4, the basal OCR in the cell lines CV0, CVND6, M, MV0, and MVND6 were 99%, 112%, 76%, 88% and 106% relative to the mean value measured in the control cell lines. To investigate which of the enzyme complexes of the respiratory chain were affected in the mutant cell lines, oligomycin (to inhibit the ATP synthase), carbonyl cyanide p-trifluoromethoxyphenylhydrazone (FCCP) (to uncouple the mitochondrial inner membrane and allow for maximum electron flux through the ETC), rotenone (to inhibit complex I), and antimycin A (to inhibit complex III) were added sequentially while measuring OCR. The difference between the basal OCR and the drug-insensitive OCR yields the amount of ATP-linked OCR, proton leak OCR, maximal OCR, reserve capacity, and non-mitochondrial OCR. As shown in Fig. 4, the ATP-linked OCR, proton leak OCR, maximal OCR, reserve capacity, and non-mitochondrial OCR in mutant cell lines expressing ND6 gene increased 37.8%, 25.3%, 21.9%, 8.4% and 39.4%, relative to the mean value measured in the mutant cell lines, respectively. In contrast, the levels of ATP-linked OCR, proton leak OCR, maximal OCR, reserve capacity, and non-mitochondrial OCR in control cell lines expressing ND6 gene ranged from 104 to 125%, relative to the mean value measured in the control cell lines, respectively.

### Increasing levels of mitochondrial ATP

**Fig. 5:**
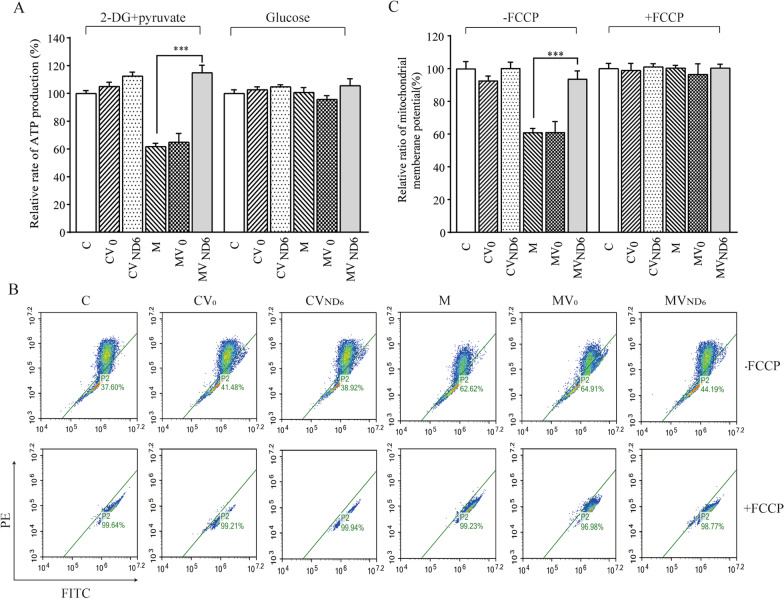
Measurements of mitochondrial ATP levels and membrane potential. **A** ATP levels among six cell lines C, CV0, CVND6, M, MV0, and MVND6 were measured using a luciferin/luciferase assay. Cells were incubated with 10 mM glucose or 5 mM 2-DG plus 5-mM pyruvate to determine ATP generation under mitochondrial ATP synthesis. Average rates of total cellular and mitochondrial ATP level per cell line and are shown. Three independent experiments were made for each cell line. **B** and **C** Mitochondrial membrane potential analysis. **B** Represented flow cytometry images of the six cell lines C, CV0, CVND6, M, MV0, and MVND6 in the presence and absence of 10 μM of FCCP. **C** The relative ratios of JC-10 fluorescence intensities at excitation/emission of 490/530 nm and 490/590 nm in the absence and presence of FCCP (**C**). Three independent experiments were made for each cell line. Graph details and symbols are explained in the legend to Fig. 2

To examine the effect of overexpression of ND6 on the oxidative phosphorylation, we measured the levels of cellular and mitochondrial ATP production using a luciferin/luciferase assay. Populations of cells were incubated in media in the presence of glucose (total cellular ATP), or 2-deoxy-D-glucose (2-DG) with pyruvate to inhibit glycolysis (mitochondrial ATP) [38]. As shown in Fig. 5A, the levels of mitochondrial ATP (the presence of pyruvate and 2-deoxy-D-glucose to inhibit the glycolysis) in the cell lines CV0, CVND6, M, MV0, and MVND6 were 105%, 113%, 62%, 65% and 115% respectively, relative to the mean values in the control cell line C. On the contrary, the overexpression of ND6 did not significantly change the levels of total cellular ATP (the presence of glucose) in the various cell lines.

### Enhanced mitochondrial membrane potential

The mitochondrial membrane potential (ΔΨm) generated by proton pumps (complexes I, III and IV) is an essential component in the process of energy storage during oxidative phosphorylation [43]. JC-10 Mitochondrial Membrane Potential Assay Kit was used to measure the ΔΨm levels in the cell lines. As shown in Fig. 5B and C, the ΔΨm levels of the cell lines CV0, CVND6, M, MV0, and MVND6 in the absence of FCCP were 93%, 100%, 61%, 61% and 94% relative to the mean values in the control cell line C, respectively. In contrast, the levels of ΔΨm in these six cell lines were comparable with those in the presence of FCCP.

### Reduced the production of mitochondrial ROS

**Fig. 6:**
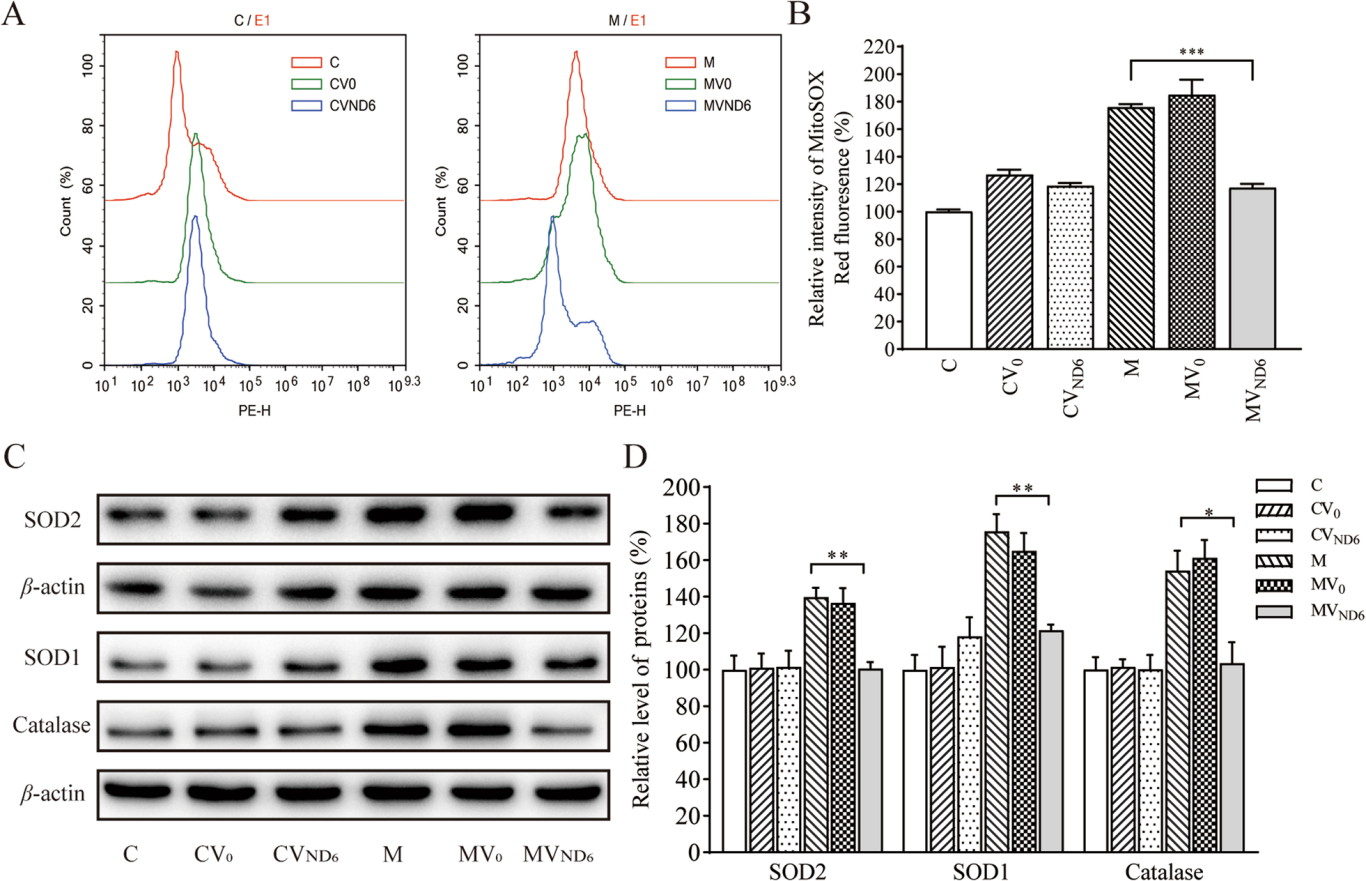
Assays for ROS production. The rates of ROS generation by mitochondria in living cells from six cell lines were analyzed by a Novocyte flow cytometer (ACEA Biosciences) using the mitochondrial superoxide indicator MitoSOX-Red (5 μM). **A** Flow cytometry histogram showing MitoSOX-Red fluorescence of various cell lines. **B** The relative ratios of intensity were calculated. The average values of three independent determinations for each cell line were shown. **C** Western blotting analysis of anti-oxidative enzymes SOD1, SOD2 and catalase in six cell lines with *β*-actin as a loading control. **D** Quantification of SOD1, SOD2 and catalase. Three independent experiments were made for each cell line. Graph details and symbols are explained in the legend to Fig. 2

Mitochondrial ROS play a critical role in physiological consequences [17, 36]. The levels of the ROS generation among these cells were measured using MitoSOX assay via flow cytometry. Geometric mean intensity was recorded to measure and delineate the rate of ROS of each sample [36, 44]. As shown in Fig. 6A and B, the levels of mitochondrial ROS production in the cybrid cell lines CV0, CVND6, M, MV0 and MVND6 were 127%, 119%, 176%, 185% and 117% relative to the mean values in the control cell line, respectively.

To determine whether the overexpression of ND6 affected the expression of antioxidant system, we examined the levels of catalase and superoxide dismutase proteins (SOD1 and SOD2) in various cell lines by Western blot analysis. As shown in Fig. 6C and D, the levels of catalase, SOD1, and SOD2 in cell line M were 154%, 176%, and 140% relative to the mean values in the control cell line C, while the levels of catalase, SOD1 and SOD2 in cell line MVND6 were104%, 122% and 100% relative to the mean values in the control cell line C. These data indicated that overexpression of ND6 in the mutant cell line decreased the levels of ROS production due to m.14484T > C mutation.

### Inhibiting apoptosis

**Fig. 7:**
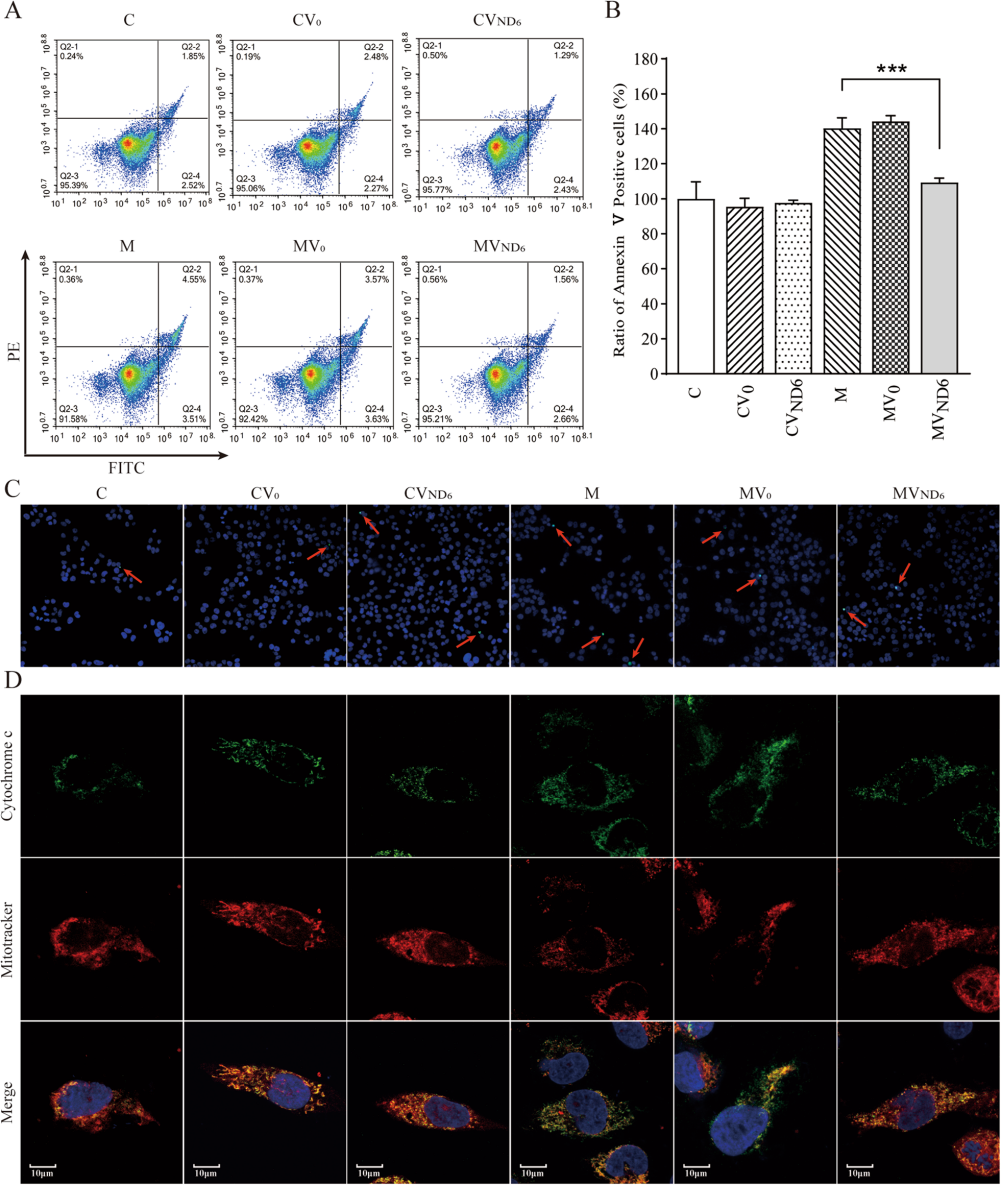
Apoptosis assays. **A** Annexin V/PI apoptosis assay by flow cytometry. Cells were harvested and stained with Annexin V and 1 μL of propidium iodide. The percentage of Annexin V-positive cells were then assessed. **B** Relative Annexin V-positive cells from various cell lines. Three independent determinations were done in each cell line. **C** TUNEL assays of the six cell lines C, CV0, CVND6, M, MV0, and MVND6. Arrows indicate death cells. **D** Immunofluorescence analysis. The distributions of cytochrome c from the six cell lines were visualized by immunofluorescent labeling with cytochrome c antibody conjugated to Alex Fluor 488 (green) and Mitotracker (red) analyzed by confocal microscopy. DAPI stained nuclei were identified by their blue fluorescence

We evaluated if the overexpression of ND6 affected the apoptotic process using six cell lines. We examined the apoptotic state of cell lines by Annexin V/PI-based flow cytometry, TUNEL, immunocytostaining and Western blot analyses. As shown in Fig. 7A and B, the average ration of Annexin V-positive cells in the cell lines CV0, CVND6, M, MV0, and MVND6 were 96%, 98%, 140%, 144%, and 109% relative to the mean values in the control cell line C. As shown in Fig. 7C, TUNEL assays revealed that cell death in the mutant cell lines M and MV0 increased as compared with the control cell line C and CV0, but overexpression of ND6 in the mutant cell line led to reductions in the cell death. In particular, there were 19 cell deaths out of 2664Total cells in the cell line C, 27 cell deaths out of 3490 total cells in the cell line CV0, 28 cell deaths out of 3273 total cells in the cell line CVND6, 114 cell deaths out of 3208 total cells in the cell line M, 69 cell deaths out of 2057 total cells in the cell line MV0, and 45 cell deaths out of 2524 total cells in the cell line MVND6. We then examined the apoptotic state of the cell lines by using immunocytostaining assays that the immunofluorescence patterns of double labeled cells with mouse monoclonal antibody specific for the cytochrome c and specific dye for mitochondria Mito-Tracker Red. As shown in Fig. 7D, overexpression of ND6 cell line MVND6 exhibited less levels of cytosolic cytochrome c, than those in the parental mutant cells M.

**Fig. 8:**
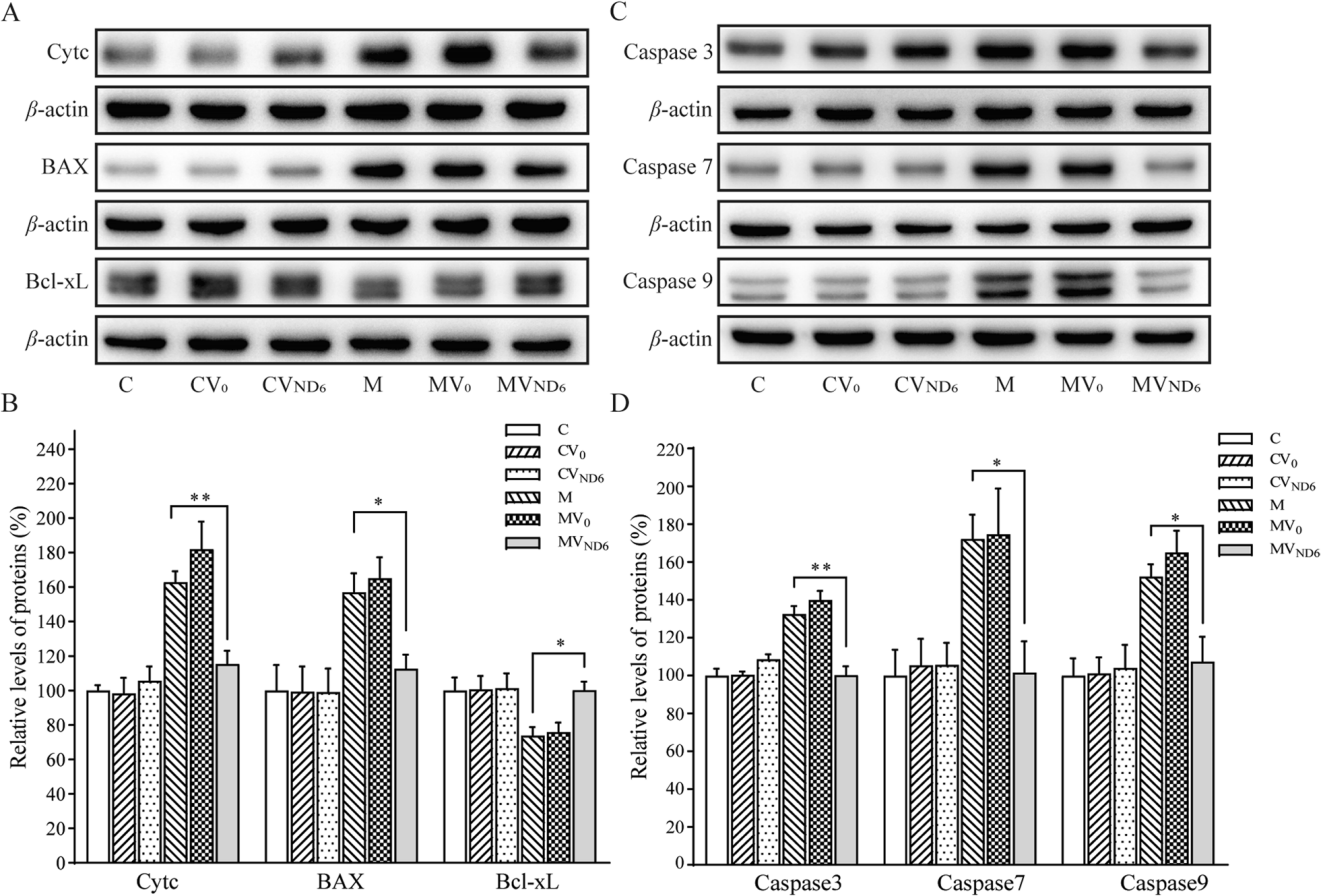
Analysis of apoptosis-associated proteins. **A** Western blotting analysis. Twenty micrograms of total cellular proteins from various cell lines were electrophoresed through a denaturing polyacrylamide gel, electroblotted, and hybridized with cytochrome c, BAX and Bcl-xL antibodies, with *β*-actin as a loading control. **B** Quantification of cytochrome c, BAX and Bcl-xL. Three independent experiments were made for each cell line. **C** Western blotting analysis of apoptosis-associated protein uncleaved caspases 3, caspases 7 and caspases 9 in six cell lines with *β*-actin as a loading control. **D** Quantification of uncleaved caspases 3, caspases 7 and caspases 9. Three independent experiments were made for each cell line. Graph details and symbols are explained in the legend to Fig. 2

The impact of ND6 overexpression on the apoptotic process was further analyzed with Western blot analysis. As shown in Fig. 8A and B, the levels of cytochrome c in the cell lines CV0, CVND6, M, MV0, and MVND6 were 98%, 106%, 163%, 182%, and 115% relative to the mean values in the control cell line C. Furthermore, we measured the levels of apoptosis-related proteins: BAX, Bcl-xL, Caspases 3, 7 and 9 in mutant and control cell lines by Western blot analysis. The levels of BAX in the cell lines CV0, CVND6, M, MV0, and MVND6 were 99%, 99%, 157%, 165%, and 112% relative to the mean values in the control cell line C, respectively, while the levels of Bcl-xL in the cell lines CV0, CVND6, M, MV0, and MVND6 were 100%, 101%, 74%, 76%, and 100% relative to the mean values in the control cell line C, respectively (Fig. 8A, B). As shown in Fig. 8C and D, the levels of caspases 3, 7 and 9 in the cell line MVND6 were significantly reduced, as compared with mutant cell line M. In particular, the levels of caspase 3, caspase 7, caspase 9 in in the cell line MVND6 were 100%, 102%, and 107%, those in the cell line M were 133%, 172%, and 152%, as compared with the parental control cell line C, respectively.

### Restoration of impaired mitophagy

**Fig. 9:**
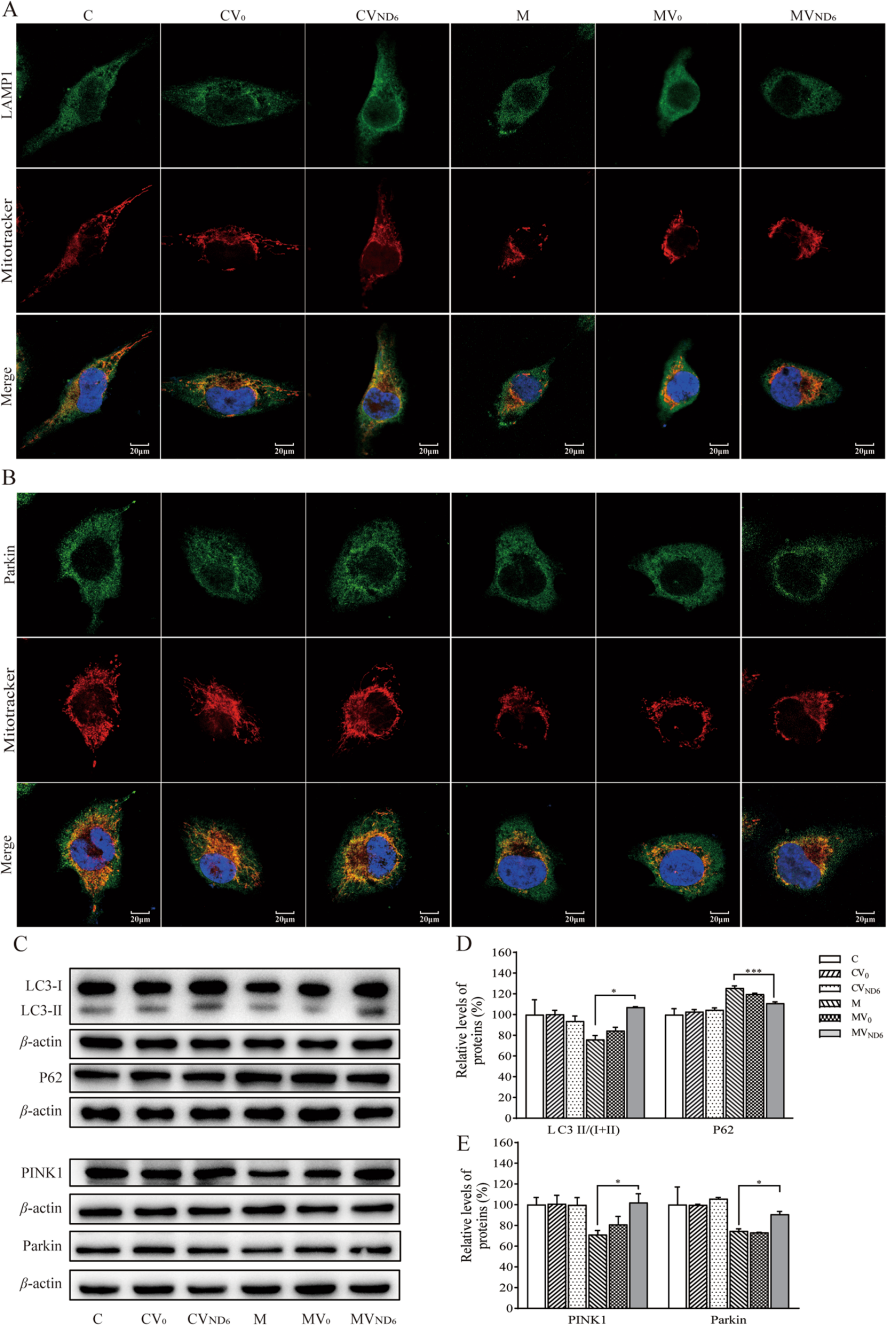
Analysis of mitophagy. **A** and **B** Immunofluorescence analysis. The distributions of LAMP1 (A) and Parkin (B) from various cell lines were visualized by immunofluorescent staining with mitochondrial dye MitoTracker (red) and labeling with LAMP1 or Parkin. Scale bars: 20 µm. **C** Western blot analysis. Twenty micrograms of total cellular proteins from various cell lines were electrophoresed through a denaturing polyacrylamide gel, electroblotted, and hybridized with LC3, P62, PINK1, Parkin antibodies, with *β*-actin as a loading control, respectively. **D** and **E** Quantification of LC3 II/(I + II), P62, PINK1 and Parkin. Three independent experiments were done for each cell line. Graph details and symbols are explained in the legend to Fig. 2

Our previous study showed that the m14484T > C mutation impaired the mitophagy [20]. We tested if the overexpression of ND6 restored impaired mitophagy due to the m.14484T > C mutation by immunocytostaining and Western blot analyses. As shown in Fig. 9A and B, the mutant cybrids expressing nucleus-version of ND6 (MVND6) exhibited increased levels of LAMP1 (lysosome-associated membrane glycoprotein1) and Parkin, as compared with those in the parental mutant cybrids M. As shown in Fig. 9C, the effect of expressing nucleus-version of ND6 on mitophagy were further assessed by Western blot analysis using four antibodies of mitophagy-related proteins [LC3 (microtubule-associated protein 1A/1B light chain 3), P62 (sequestosome 1), PINK1 (mitochondrial serine/threonine-protein kinase) and Parkin (E3 ubiquitin ligase)] [47, 48]. As shown in Fig. 9D–E, the average levels of LC3II/I + II, P62, PINK1 and Parkin in the mutant cybrids expressing nucleus-version of ND6 (MVND6) were significantly elevated, as compared with these in the parental mutant cybrids (M) and comparable with those in the parental control cybrids C.

## Discussion

Despite substantial progress in the development of allotopic expression of ND4 gene for therapy of the LHON, some challenges remain to be overcome to accelerate the successful application of this technique [30–33]. In the present study, we investigated the mechanism underlying the allotopic expression of mitochondrial ND6 transgene for correcting the mitochondrial dysfunctions due to the LHON-associated m.14484T > C mutation. For this purpose, the control and mutant stable transfectants were generated by inserting a synthetic ND6 compatible with the universal genetic code in a pCDH-puro vector and importing it into mitochondria by adding a mitochondrial targeting sequence, which produced a stable gene-expressed signature that persists even after mitochondrial repopulation. In the present investigation, we were successful in expressing and targeting ND6 allotopically. The gene product seems to be correctly processed and imported into the mitochondria. In fact, the m.14484T > C (p.M64V) mutation altered structural and function of complex I. As a result, the m.14484T > C mutation-induced complex I deficiency led to the decreases in mitochondrial ATP production and membrane potential, increases in production of reactive oxygen species, promoted apoptosis and impaired mitophagy [20]. In the present investigation, we demonstrated that the allotopic expression of ND6 gene in the cybrids bearing the m.14484T > C mutation restored the complex I deficiency, raised mitochondrial ATP production and membrane potential, reduced the ROS production, apoptosis and restored impaired mitophagy (Table 1).

**Table 1:** Summary of biochemical data

		C	CV_0_	CV_ND6_	M	MV_0_	MV_ND6_
Western blot analysis	ND6	100.0 ± 10.3	102.2 ± 6.8	120.4 ± 13.4	73.5 ± 2.0	74.8 ± 6.7	101.4 ± 4.9
	ND4L	100.0 ± 2.6	98.5 ± 5.3	113.4 ± 16.7	56.0 ± 6.4	55.8 ± 23.3	103.9 ± 5.8
	ND1	100.0 ± 9.1	102.2 ± 11.3	103.6 ± 12.6	77.4 ± 9.7	82.0 ± 11.3	110.5 ± 14.9
In-gel activity of complexes	Complex I	100.0 ± 5.1	101.3 ± 4.2	103.3 ± 6.2	78.4 ± 5.2	80.1 ± 5.9	101.0 ± 4.3
	Complex IV	100.0 ± 5.1	102.1 ± 5.9	102.6 ± 6.6	104.6 ± 4.2	102.1 ± 3.7	99.5 ± 6.4
Steady levels of complexes	Complex I	100.0 ± 2.9	101.8 ± 3.3	100.2 ± 2.5	81.9 ± 3.9	81.0 ± 4.3	101.7 ± 6.6
	Complex II	100.0 ± 14.7	99.5 ± 15.3	100.3 ± 14.4	101.1 ± 13.7	101.7 ± 13.5	100.6 ± 10.8
	Complex IV	100.0 ± 13.1	101.4 ± 5.9	102.5 ± 6.3	99.1 ± 4.7	103.3 ± 7.9	100.1 ± 17.1
Membrane potential	- FCCP	100.0 ± 4.3	92.7 ± 2.7	100.3 ± 3.7	61.0 ± 2.4	61.2 ± 6.6	93.7 ± 4.8
ATP levels	2DG + pyruvate	100 ± 2.1	105.1 ± 3.0	112.6 ± 2.8	61.8 ± 2.3	64.9 ± 6.3	115.0 ± 5.4
	Glucose	100.0 ± 2.6	102.7 ± 2.0	104.8 ± 1.3	100.7 ± 3.5	95.7 ± 2.7	105.6 ± 5.0
OCR	Basal	100.0 ± 3.5	99.4 ± 6.9	112.2 ± 5.5	76.1 ± 10.4	87.8 ± 5.7	106.4 ± 2.6
	ATP-Link	100.0 ± 6.1	102.0 ± 35.6	125.7 ± 5.8	62.7 ± 21.6	65.5 ± 36.8	100.5 ± 3.2
	Proton-Link	100.0 ± 7.6	97.7 ± 14.8	103.5 ± 10.8	84.9 ± 13.4	102.3 ± 19.6	110.2 ± 2.6
	Maximal	100.0 ± 8.4	101.0 ± 6.9	109.6 ± 4.8	75.7 ± 16.3	87.6 ± 7.5	97.6 ± 3.5
	Reserve Capacity	100.0 ± 19.0	103.6 ± 9.2	105.4 ± 9.9	75.1 ± 25.8	87.4 ± 11.3	83.5 ± 13.5
	Non-mitochondrial	100.0 ± 6.0	84.2 ± 5.2	104.4 ± 17.9	79.6 ± 8.4	80.6 ± 4.3	119.0 ± 2.9
Mitochondrial ROS		100.0 ± 1.5	126.8 ± 3.7	118.7 ± 2.0	176.0 ± 2.0	185.0 ± 10.9	117.2 ± 2.9
Annexin V/PI apoptosis		100.0 ± 16.7	95.5 ± 8.3	97.7 ± 2.5	140.3 ± 10.5	144.2 ± 5.9	109.3 ± 4.4
Anti-oxidative enzymes	SOD2	100.0 ± 13.3	101.1 ± 13.4	101.6 ± 15.1	139.7 ± 9.0	136.6 ± 14.1	100.5 ± 6.5
	SOD1	100.0 ± 14.0	101.1 ± 19.2	118.3 ± 18.1	175.8 ± 16.5	165.2 ± 16.9	121.6 ± 5.5
	Catalase	100.0 ± 12.0	101.7 ± 7.1	100.2 ± 13.7	154.2 ± 19.0	161.3 ± 16.8	103.6 ± 19.9
Apoptosis-associated proteins	Cytochrome C	100.0 ± 5.3	98.2 ± 15.8	105.7 ± 14.2	162.8 ± 11.0	181.9 ± 27.9	115.3 ± 13.3
	BAX	100.0 ± 25.7	99.2 ± 25.4	99.1 ± 23.4	156.9 ± 19.3	165.1 ± 21.1	112.5 ± 14.4
	Bcl-xL	100.0 ± 13	100.6 ± 13.4	101.4 ± 14.8	73.8 ± 8.7	75.8 ± 9.7	100.1 ± 8.6
	Caspase 3	100.0 ± 6.5	100.3 ± 3.3	108.6 ± 4.7	132.5 ± 7.4	140.0 ± 8.4	100.2 ± 8.2
	Caspase 7	100.0 ± 23.6	105.4 ± 24.2	105.7 ± 20.3	172.2 ± 22.3	174.6 ± 42.2	101.6 ± 28.5
	Caspase 9	100.0 ± 15.9	101.1 ± 14.8	103.9 ± 21.4	152.4 ± 11.2	165.1 ± 19.8	107.3 ± 22.9
Mitophagy-associated proteins	LC3	100.0 ± 24.7	100.2 ± 6.8	93.7 ± 8.5	75.9 ± 6.9	84.5 ± 5.6	107.1 ± 0.7
	P62	100.0 ± 9.9	102.8 ± 3.7	104.5 ± 3.3	125.6 ± 3.6	119.7 ± 1.7	110.9 ± 2.3
	PINK1	100.0 ± 12.0	100.8 ± 14.4	99.8 ± 12.3	71.1 ± 7.0	81.0 ± 13.3	102.1 ± 14.9
	Parkin	100.0 ± 29.6	99.8 ± 1.1	105.8 ± 2.2	74.5 ± 3.7	73.3 ± 1.5	90.7 ± 4.9

Strikingly, the allotopic expression of nucleus-version of ND6 in the mutant cybrids carrying the m14484T > C mutation raised 28%, 48% levels of ND6, ND4L and 33% in ND1, respectively, but not those in the control cybrids lacking the mutation. The increasing levels of ND1 and ND4L are likely due to the improved ability of ND6 interact with ND4L or ND1 in the complex I [20, 21]. By contrast, the overexpression of ND4 or ND1 gene in the mutant cybrids only elevated the levels of ND4 or ND1, respectively [31, 32]. The fact that the expression of nucleus-version of ND6 did not elevate the level of ND6 in the wild-type cybrids indicated that this ND6 level appeared to be the maximum threshold level to maintain the normal function, as in the case of no change in the level of aminoacylated tRNA^Leu(UUR)^ in the wild-type cybrids by overexpression of LARS2 [49].

In this study, the mutant cybrids expressing the wild type nucleus-version of ND6 exhibited 20%-23% increases in the assembly and activity of complex I and basal OCR, as compared with the parental mutant cell line. It was very likely that the allotopic expression of wild type nucleus-version of ND6 substituted mutated ND6, stabilized interactions between ND6 and ND1 or ND4L, increased number of corrected Complex I assembly necessary to improve OXPHOS function. As a result, the elevating activities of respiratory chain complexes caused by overexpression of ND6 yielded ~ 53% and ~ 33% increases in the levels of mitochondrial ATP and ΔΨm in the mutant cell line bearing the m.14484T > C mutation. Indeed, ΔΨm in the cell lines reflects the pumping of hydrogen ions across the inner membrane during the process of electron transport and oxidative phosphorylation [43]. The improvement of both OXPHOS and ΔΨm would reduce the production of ROS [36]. In this investigation, mutant cell lines with overexpression of ND6 exhibited marked reductions in the levels of mitochondrial ROS and three antioxidant enzymes, SOD2 in the mitochondrion and SOD1 and catalase in the cytosol catalase. The lower production of ROS can reduce a vicious cycle of oxidative stress in the mitochondria, thereby decreasing the damage of mitochondrial and cellular proteins, lipids and nuclear acids [50].

Mitochondrial dysfunctions caused by LHON-associated mtDNA mutations impaired the cell viability and affected the apoptotic process as well as mitophagy [19, 20, 45, 51–53]. In particular, mutant cybrids bearing the m.14484T > C mutation exhibited increasing ratio of Annexin V-positive cells and elevated releases of cytochrome c into cytosol than those in control cybrids [20]. In the present study, we demonstrated that the overexpression of ND6 suppressed the m.14484T > C mutation-induced the impairment of apoptosis. Lines of evidence from Annexin V/PI-based flow cytometry, TUNEL and immunocytostaining assays indicated much less apoptosis in the mutant cybrids with overexpression of ND6 than those in the parental mutant cybrids. These were further supported by decreasing levels in cytochrome c, and BAX which mediate cell death by apoptosis [54], raising expressions of Bcl-xL which has anti-apoptotic activity [55], and rescuing levels of apoptosis activated proteins: caspases 7, 9, and 3 in the mutant cybrids with overexpression of ND6, as compared with than those in the parental mutant cybrids [56, 57]. Notably, the levels of apoptosis in the mutant cybrids with overexpression of ND6 were comparable with those in the control cybrids. Furthermore, our previous study showed that the m.14484T > C mutation impaired the mitophagy [20]. In this study, we demonstrated that the overexpression of ND6 restored impaired mitophagy due to the m.14484T > C mutation by immunocytostaining and Western blot analyses. In particular, the average levels of LC3II/I + II, P62, PINK1 and Parkin in the mutant cybrids expressing nucleus-version of ND6 were significantly elevated, as compared with these in the parental mutant cybrids. These data indicated that overexpression of human ND6 reversed the abnormal cell apoptosis and mitophagy which caused by m.14484T > C mutation. Therefore, the overexpression of ND6 in mutant cells may reprogram energy metabolism and prevent the dysfunction or death of retinal ganglion cells due to the m.14484T > C mutation [58].

## Conclusion

In this investigation, we demonstrated that allotopic expression of human ND6 restored complex I, apoptosis and mitophagy deficiencies caused by LHON-linked m.14484T > C mutation. The restoration of m.14484T > C mutation-induced mitochondrial dysfunctions by overexpression of ND6 is a step toward therapeutic interventions for LHON and other mitochondrial diseases.

## Data Availability

Representative experiments are shown in the figures and additional materials. For any additional information, please contact the corresponding author.
